# Label-Free Quantitative Proteome Analysis Reveals the Underlying Mechanisms of Grain Nuclear Proteins Involved in Wheat Water-Deficit Response

**DOI:** 10.3389/fpls.2021.748487

**Published:** 2021-10-25

**Authors:** Tingting Li, Dong Zhu, Zhisheng Han, Junwei Zhang, Ming Zhang, Yueming Yan

**Affiliations:** ^1^Beijing Key Laboratory of Plant Gene Resources and Biotechnology for Carbon Reduction and Environmental Improvement, College of Life Sciences, Capital Normal University, Beijing, China; ^2^College of Agricultural and Biological Engineering (College of Tree Peony), Heze University, Heze, China

**Keywords:** wheat, developing grains, water deficit, nuclear proteome, label-free quantitation

## Abstract

In this study, we performed the first nuclear proteome analysis of wheat developing grains under water deficit by using a label-free based quantitative proteomic approach. In total, we identified 625 unique proteins as differentially accumulated proteins (DAPs), of which 398 DAPs were predicted to be localized in nucleus. Under water deficit, 146 DAPs were up-regulated and mainly involved in the stress response and oxidation-reduction process, while 252 were down-regulated and mainly participated in translation, the cellular amino metabolic process, and the oxidation-reduction process. The *cis*-acting elements analysis of the key nuclear DAPs encoding genes demonstrated that most of these genes contained the same *cis*-acting elements in the promoter region, mainly including ABRE involved in abscisic acid response, antioxidant response element, MYB responsive to drought regulation and MYC responsive to early drought. The *cis*-acting elements related to environmental stress and hormones response were relatively abundant. The transcription expression profiling of the nuclear up-regulated DAPs encoding genes under different organs, developmental stages and abiotic stresses was further detected by RNA-seq and Real-time quantitative polymerase chain reaction, and more than 50% of these genes showed consistency between transcription and translation expression. Finally, we proposed a putative synergistic responsive network of wheat nuclear proteome to water deficit, revealing the underlying mechanisms of wheat grain nuclear proteome in response to water deficit.

## Introduction

As an allohexaploid species and the product of intensive breeding and long-term artificial domestication, wheat (*Triticum aestivum* L., 2*n* = 6*x* = 42, AABBDD) is the second leading cereal crop widely cultivated in the world. Wheat grain flour can be used to make various special foods such as breads, noodles, cakes, and biscuits (Shewry, 2009). The mature wheat grains contain a variety of nutrients including starch and storage protein that are rapidly synthesized and accumulated after flowering (Guo et al., 2012; Liu et al., 2012). Their compositions and properties play important roles in wheat yield and quality formation. In particular, gliadins, and glutenins are the major storage protein of endosperm and confer dough viscoelasticity that is closely related to bread making quality (Shewry, 2009; Yan et al., 2009; Xie et al., 2010).

Wheat often encounters various biotic and abiotic stresses such as drought, high temperature, and insect pests during growth and development. Drought stress that has occurred at wheat grain developing stages severely affects protein and starch biosynthesis, leading to a significant reduction in grain weight and yield (Dupont and Altenbach, 2003; Duan et al., 2020). In the face of drought and other adverse stress events, plants have developed various mechanisms for survival. The signal transduction is a key mechanism for perceiving and transmitting water stress signals so that adaptive responses are activated (Zhu, 2002). Drought also induces the formation of plant hormones such as abscisic acid (ABA), jasmonate and ethylene to promote the production of reactive oxygen species (ROS). Plant signal transduction in response to drought stress includes an ABA-dependent osmotic stress signal transduction, MAPKKK signal cascade transduction pathway and an ROS scavenging response pathway. In addition, plants can rid themselves of excessive toxic substances such as ROS caused by drought, as well as protect their membrane system by activating the enzymatic and non-enzymatic scavenging systems (Pandey et al., 2008; Ge et al., 2012). With the recent development of wheat genomics, proteomics has made great progress in revealing plant synergistic response mechanisms to drought stress. To date, proteomic responses to drought stress of wheat’s different organs have been widely studied, including seedling roots and leaves (Cheng et al., 2015; Hao et al., 2015), grain development (Ge et al., 2012; Deng et al., 2018; Duan et al., 2020), flag leaves (Deng et al., 2018; Zhu et al., 2020), and glume and awn (Deng et al., 2019). Various protein post-translational modifications also participate in regulating drought responses such as phosphorylation and acetylation modifications (Zhang et al., 2014a, b; Zhu et al., 2018). In recent years, subcellular proteomics has been developed to decipher different organelle proteins involved in abiotic stress defense such as endoplasmic reticulum, plasma membrane, chloroplast, and mitochondria (Wang and Komatsu, 2016; Zhang et al., 2021; Zhu et al., 2021).

Considerable transcriptome and proteome work showed that wheat developing grains contained a large number of genes and proteins that are involved in biotic and abiotic stress responses (Gao et al., 2009; Ge et al., 2012; Yu et al., 2016). The nucleus is a highly ordered subcellular organization structure of core area of a cell. It contains biological genetic information that regulates gene expression and stress responses (Pandey et al., 2008). At present, studies on nuclear proteome involved in plant growth and stress response have been performed in some plant species, including Arabidopsis (Palm et al., 2016), rice (Choudhary et al., 2009), soybean (Yin and Komatsu, 2015; Wang and Komatsu, 2016), chickpea (Pandey et al., 2008; Kumar et al., 2014), and *Medicago truncatula* (Repetto et al., 2008). In wheat, only a few studies on nuclear proteome have been reported so far. Bonnot et al. (2015) used nano liquid chromatography-tandem mass spectrometry to identify 387 nuclear protein spots in the developing wheat grains. The researchers found that some proteins were involved in regulating transcription such as HMG1/2-like protein and histone deacetylase HDAC2 with abundant expression in the phase between cellularization to grain-filling. Bancel et al. (2015) analyzed the nuclear proteome of two wheat varieties at two developmental stages by mass spectrometry. The study found some differently expressed proteins between species and developmental stages, and many these proteins may be involved in regulating protein synthesis in grains. However, wheat nuclear proteome responses to abiotic stress during grain development remain unknown.

Over the past several decades, there have been great developments in mass spectrometry technology. As a major breakthrough, label-free techniques enable protein quantification relative to the levels of corresponding peptides in a sample and greatly improve the sensitivity for protein detection, especially for the detection of low abundance proteins (Bantscheff et al., 2007). In this study, we performed the first label-free quantitative nuclear proteome analysis to uncover the underlying drought response and defense mechanisms of wheat nuclear proteins in developing wheat grains. Our results revealed a synergistic response network of wheat nuclear proteins under field water-deficit conditions, thereby providing new insights into the mechanisms of plant abiotic stress response.

## Materials and Methods

### Plant Material, Field Trial and Water-Deficit Treatment

Plant material used in this study was Chinese elite winter wheat cultivar Zhongmai 175. This cultivar has many advantages such as better adaptation, high yield and superior noodle quality widely cultivated in the north wheat production area of China in recent years (Li et al., 2016). Field trial was conducted at the experimental station of China Agricultural University, Beijing during 2019–2020 growing season, including a control group with well-watered irrigation and a water-deficit treatment group without irrigation after flowering. The traditional flood irrigation with 75 mm at both jointing and flowering stages was set as well-watered irrigation while rain-fed region without artificial water irrigation was used as no irrigation group. Three biological replicates were performed for each treatment (each plot was 4 m × 9 m with row spaced at 0.16 m). To minimize the interference from adjacent plots, 1-m-wide zone between plots was set as an unirrigated zone. According to the previous study, the stage of 9–18 day post-anthesis (DPA) is the critical period of starch synthesis and accumulation during grain filling in response to water deficit (Yang et al., 2004). Thus, the developing grain samples from 15 DPA were collected for later analysis in this study.

### Isolation and Purification of Cell Nucleus From Wheat Developing Grains

Isolation and purification of grain cell nuclear were based on Bancel et al. (2015) with modifications. The developing grain samples about 2 *g* from 15 DPA were grounded into powder with liquid nitrogen. The powder was thoroughly mixed in 8 mL extraction buffer with 20 mM HEPES-KOH (pH 7.0), 5 mM MgCl_2_, 10 mM 2-mercaptoethanol, 0.5 mM PMSF, and 0.1% (v/v) phosphatase inhibitor (Sigma-Aldrich). After filtering through a two-layer Miracloth filter (Calbiochem, pore size 25 μm) for removing cell debris, the homogenate was transferred into a 15 mL centrifuge tube and more extraction buffer was added with a final volume of 12 mL containing 0.5% (v/v) Triton X-100 for lysing membranes, and then incubated at 4°C for 15 min. After centrifugation at 1,000 *g* and 4°C for 10 min, the supernatant was collected, and the precipitation was washed four times with extraction buffer. Meanwhile, the supernatant was successively collected in five-time extractions and, respectively, marked as sample S1–S5.

The crude extracts of cell nucleus were resuspended in 4 mL extraction buffer and placed on the top of a stepwise Percoll gradient (4 mL 30% Percoll solution and 4 mL 80% Percoll solution). After centrifuging at 930 *g* and 4°C for 30 min, the nucleus floating at the interface of 30 and 80% liquid layers were collected and washed twice with 4 mL extraction buffer. The nuclear pellets were resuspended in PBS buffer (1.47 mM KH_2_PO_4_, 4.3 mM Na_2_HPO_4_⋅2H_2_O, 2.7 mM KCl, and 137 mM NaCl), and an aliquot was stained in 0.1 μg/mL Hoechst 33,258 fluorescent dye solution for 20 min. Finally, the nuclear signals were detected by confocal microscope (Leica TCS SP5, Germany) to evaluate nuclear integrity and enrichment flux.

### Nuclear Protein Extraction

The isolated nuclear samples were resuspended in 500 μL of TRI reagent (Sigma-Aldrich) and incubated at room temperature for 5 min. Then, 50 μL of 1-bromo-3-chloropropane was added to the mixture and vortexed for 15 s. After incubation at room temperature for 15 min and centrifugation at 12,000 *g* and 4°C for 15 min, RNA located on the upper phase was removed. To precipitate DNA, 150 μL of 95% (v/v) ethanol was added, and incubated at room temperature for 3 min. The mixture was centrifuged at 2,000 *g* and 4°C for 5 min and the supernatant was collected. Then, 750 μL of 2-propanol was added and incubated at room temperature for 10 min. After centrifugation at 12,000 *g* and 4°C for 10 min, the protein pellets were collected and washed three times with 95% (v/v) ethanol. Freeze drying was performed and then the protein samples were kept at -80°C for later use.

### Trypsin Digestion

Dithiothreitol was added to dried nuclear proteins with a final concentration of 5 mM and incubated at 56°C for 30 min. After that, proper amount of iodoacetamide was added to the protein mix with a final concentration of 11 mM, and further incubated at room temperature for 15 min in darkness. Subsequently, the protein samples were diluted by adding 100 mM NH_4_HCO_3_ to a urea concentration less than 2 M and digested with trypsin buffer at a final enzyme/protein ratio of 1:50 (w/w) at 37°C overnight. Then, second digestion was performed with trypsin buffer at a final enzyme/protein ratio of 1:100 (w/w) for 4 h.

### UPLC Fractionation

The digested peptides were fractionated through reversed-phase high performance liquid chromatogram by using a chromatographic column (Agilent 300 Extend C18) with an internal diameter of 4.6 mm and a particle size of 5 μm. The peptides were eluted with gradient acetonitrile (8–32%; NaHCO_3_ buffer, pH 9) and collected for later analysis.

### NanoLC-MS/MS Analysis

The grain nuclear proteome identification was performed by using EASY-nLC 1000 (Thermo/Finnigan, Sanjose, CA, United States) according to the recent report (Zhang et al., 2021).

### Sequence Database Search and Data Analysis

The resulting MS/MS data were searched using Maxquant search engine (v.1.5.2.8) against Uniprot *T. aestivum* (taxonomy: 4565 143 146 sequences). Trypsin/P was specified as cleavage enzyme allowing up to 2 missing cleavages. Mass precursor tolerance was set to 5 ppm, and MS/MS tolerance was set to 0.02 Da. False positive rate was adjusted to <1%, and minimum score for peptides was set to >40. In quantitative analysis, the quantitative values of each sample were obtained via LFQ intensity in three replicates. The ratio of the mean LFQ intensity between the two samples represents the protein fold-change value. LFQ intensity was taken as log2 transform, and then used to calculate the significant *p*-value of differential abundance between two samples. The *p*-value was calculated by using two-tailed test when the protein was quantified three times in the two compared samples. And, the available quantitative proteins were regarded as differentially accumulated proteins (DAPs) with at least two-fold change (Student’s *t*-test, *p* < 0.05) based on Bostanci et al. (2018).

### Western Blotting

The extracted nuclear proteins (N) and the five supernatant samples (S1–S5) collected during the washing process of nuclear precipitation were separated by SDS-PAGE gel, and then transferred to PVDF membrane (GE, United States) using a wet transblot system (Bio-Rad, United States). The PVDF membrane was incubated in blocking buffering for 1 h at room temperature, and then further incubated overnight at 4°C with polyclonal antibody at recommend dilution of application example. After washing three times with PBST buffer, the PVDF membrane was incubated with horseradish peroxidase-conjugated goat anti rabbit IgG at 1:5000 dilution for 1 h at room temperature. The signal was visualized by using ECL plus Western blotting detection kit (GE Healthcare Bio-Sciences AB, Uppsala, Sweden) based on the manufacturer’s instructions.

### Subcellular Localization Prediction and Verification of Nuclear Proteins

The subcellular localization of the identified DAPs were predicted according to WoLF PSORT^1^, CELLO^2^, Plant-mPLoc^3^, and UniProtKB^4^. To verify the predicted results of subcellular localization, the full-length coding sequences of the selected DAPs encoding genes were amplified by PCR and ligated into pSAT1-GFP-N (Pe3449) vector containing green fluorescent protein (GFP) gene. The recombinant plasmid DNA and pSAT1-GFP-N (Pe3449) empty control were transformed into wheat protoplasts based on Zou et al. (2020). After incubating overnight at 23°C in the dark, the GFP signal was observed by using a confocal laser scanning microscope (Leica TCS SP5, Germany).

### Functional Annotation

Gene Ontology (GO) annotation of the nuclear proteins was conducted by using AgBase version 2.00, including molecular function, cellular components, and biological functions.

### Detection of the *Cis*-Acting Elements in the Genes Encoding the Nuclear Differentially Accumulated Proteins

The gene ID of DAP was reunified into IWGSC gene ID. The *cis*-acting elements in in the 1,500 bp promoter region of the nuclear protein encoding gene were detected by PlantCARE^5^. Genome database (IWGSC RefSeqv1.1) was used to search the sequences of these nuclear proteins encoding genes with a coverage rate of 94% from GRAMENE^6^.

### Transcriptional Expression Analysis by RNA-seq

The transcriptional expression profiling of the nuclear DAPs encoding genes was analyzed using the publicly available bread wheat RNA-seq database, which included expression data from different grain developmental stages, and various stress treatments. RNA-seq data for the nuclear DAPs encoding genes in wheat grains were derived from expVIP^7^.

### RNA Extraction and Real-Time Quantitative Polymerase Chain Reaction

To detect the dynamic transcriptional expression changes of the key nuclear DAPs encoding genes under water-deficit condition, total RNA from three grain developmental stages (10, 15, and 20 DPA) was extracted using TRIZOL Reagent according to the manufacturer’s instructions. Genomic DNA removal and the reverse transcription reactions were executed by using Prime ScriptRT reagent Kit with gDNA Eraser (TaKaRa, Japan). Gene-specific primers were designed using the online tool Primer 3 Plus^8^. Ubiquitin was used as the reference for normalization. Real-time quantitative polymerase chain reaction (qRT-PCR) was conducted using CFX96 Real-Time PCRD detection System (Bio-Rad) according to Yu et al. (2016). All data were analyzed with CFX Manager Software (Bio-Rad).

## Results

### Nuclear Isolation and Quality Assessment of the Grain Nuclear Proteins

To evaluate the integrity of the isolated grain cell nucleus, we used Hoechst staining to detect the morphology of the nucleus extracted from wheat developing grains at 15 DPA (Supplementary Figure 1A). The results showed that the average diameter of the nucleus was approximately 5–10 μm, and most were complete. This means that the isolated cell nucleus had better integrity. However, when observed under a bright field environment (Supplementary Figure 1B), some substances like starch granules and cell debris were stained with fluorescent dye. This could lead the nuclear proteins to become contaminated.

**FIGURE 1:**
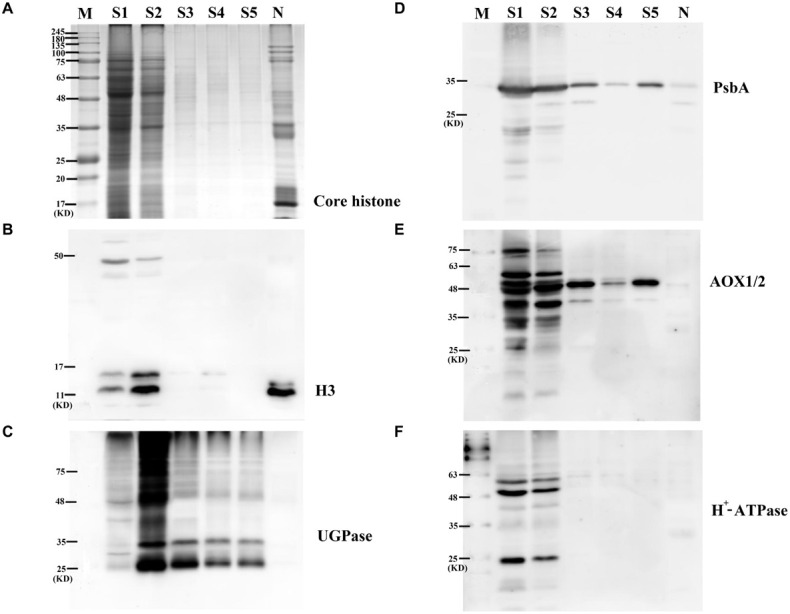
Enrichment and purity assessment of nuclear proteome from wheat developing grains. **(A)** Analytical 1-D electrophoresis profile (12.5% SDS-PAGE, CBB staining) of protein content in different stages of nuclei enrichment progress. **(B)** Immunoblot analysis with anti-histone H3. **(C)** Immunoblot analysis with anti-vacuolar UGP. **(D)** Immunoblot analysis anti-photosystem PsbA. **(E)** Immunoblot analysis with anti-AOX. **(F)** Immunoblot analysis with anti-H^+^-ATPase. S1–S5, five supernatant samples (S1–S5) collected during the washing process of nuclear precipitation; N, extracted nuclei proteins.

TRI Reagent was used to extract grain nuclear proteins from the enriched nuclear components. To estimate the efficiency of nuclear protein extraction, five supernatant samples (S1–S5) collected during the washing process of nuclear precipitation were separated by SDS-PAGE. Coomassie blue staining showed that no significant amount of proteins in S3–S5 was present, indicating that samples S1 and S2 can extract most of the nuclear proteins. As a nuclear organelle marker protein, the core histone was detectable in the nuclear proteome fractions (Figure 1A). Western blotting allowed us to further examine the purity of nuclear protein fractions by using specific antibodies against marker proteins from different organelles (Figures 1B–F), including histone H3 (H3) from cell nuclei, photosystem II reaction center protein D1 (PsbA) from chloroplasts, plant alternative oxidase 1 and 2 (AOX1/2) from mitochondria, cytoplasm UDP-glucose pyrophosphorylase from cytoplasm, and plasma membrane H^+^-ATPase (H^+^-ATPase) from plasma membrane. We detected the extremely strong nuclear specific protein H3 band and the weak chloroplast specific protein PsbA band in the nuclear protein fractions of wheat developing grains while the remaining antibodies did not show clear protein bands. These results indicated that the extracted grain cell nuclear proteins had a high purity without clear cross-contamination.

### Nuclear Proteome Response to Water Deficit

**FIGURE 2:**
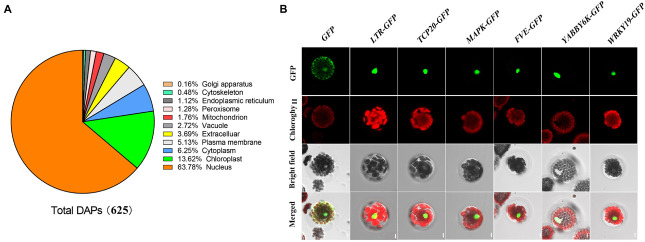
Subcellular localization of differentially accumulated proteins identified under water deficit. **(A)** Subcellular localization of differentially accumulated proteins in wheat developing grains under drought stress. **(B)** Subcellular localization of six reprehensive proteins by wheat protoplast transformation. GFP, GFP fluorescence; Chlorophyll, chlorophyll autofluorescence; Bright field, the field of bright light; Merged, merged GFP fluorescence, chlorophyll autofluorescence, and the field of bright light. LTR, low temperature-responsive RNA-binding protein; TCP20, TCP20 transcription factors; MAPK, mitogen-activated protein kinase; FVE, WD-40 repeat-containing protein; YABBY6, transcription factor YABBY6; and WRKY, transcription factor WRKY.

Label-free quantitative nuclear proteomic analysis of wheat developing grains under control and water-deficit treatment groups identified 193,026 peptides (Supplementary Table 1), corresponding to 5,850 unique proteins with a high confidence (Supplementary Table 2). Among them, we determined 625 DAPs with at least two-fold differences under water deficit (Supplementary Table 3). Based on the subcellular localization prediction by the four databases of UniProt KB, CELLO, WoLF PSORT, and Plant-mPLoc, a total of 398 DAPs accounting for 63.78% were localized in nucleus (Figure 2A and Supplementary Table 3). We predicted that the remaining DAPs would localize in different organelles, mainly including chloroplast (13.62%), cytoplasm (6.25%), plasma membrane (5.13%), extracellular (3.69%), and vacuole (2.72%). These results are generally consistent with previously reports on wheat grain nuclear proteome analysis (Bancel et al., 2015; Bonnot et al., 2015).

To verify the prediction results of the subcellular localization, we selected six DAPs predicted in the cell nucleus for further subcellular localization assay through protein transient expression in wheat protoplast, including WD-40 repeat-containing protein (FVE), transcription factor TCP20 (TCP20), mitogen-activated protein kinase (MAPK), low temperature-responsive RNA-binding protein (LTR) and transcription factors YABBY6 (YABBY6) and WRKY transcription factor 19 isoform X1 (WRKY19). We cloned the encoding sequences of these DAPs encoding genes using specific PCR primers (Supplementary Table 4), and then recombined them into pSAT1-GFP-N (Pe3449) vector carrying the GFP encoding gene. We transformed the vectors connected with the target genes into wheat protoplast for transient expression, and then observed them through a confocal microscope. The results showed that strong signals of these six DAPs occurred only in the nucleus (Figure 2B), confirming that they were all localized in the cell nucleus. These results were consistent with the website localization predictions (Supplementary Table 3).

**FIGURE 3:**
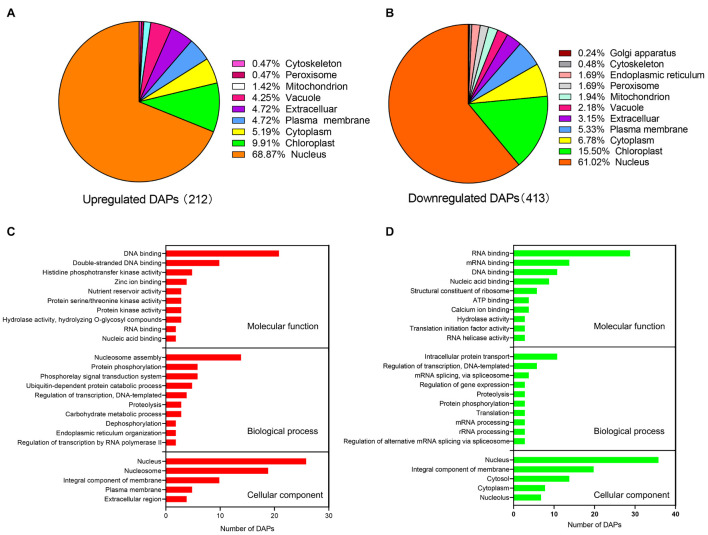
Subcellular localization and GO annotation of differentially accumulated proteins (DAPs) identified from wheat nucleus under water deficit. **(A)** Subcellular localization of significantly up-regulated nuclear proteins. **(B)** Subcellular localization of significantly down-regulated nuclear proteins. **(C)** GO annotation of significantly up-regulated proteins. **(D)** GO annotation of significantly down-regulated proteins.

Among 398 nucleus-subcellular DAPs, the abundance of 146 and 252 proteins under water deficit condition were up-regulated and down-regulated, respectively, (Figures 3A,B). To further understand the characteristics and potential roles of these DAPs in response to water deficit, we performed analyses of the GO functional classification according to their biological process, molecular function and cellular component annotation (Figures 3C,D and Supplementary Table 5). Based on our molecular function analysis, the up-regulated DAPs were mainly associated with DNA binding, histidine phosphotransfer kinase activity, while the down-regulated DAPs were mainly involved in RNA binding, DNA binding, nucleic acid binding and structure constituent of ribosome. The results of biological process analysis showed that the up-regulated DAPs were involved in nucleosome assembly, protein phosphorylation, and the phosphorelay signal transduction system. The down-regulated DAPs were associated with intracellular protein transport, regulation of transcription, mRNA splicing and regulating gene expression. In terms of the cellular component analysis, the up-regulated DAPs were mainly distributed in nucleus and nucleosome, while the down-regulated DAPs were mainly distributed in nucleus and integral component of membrane (Figures 3C,D).

### Main *Cis*-Acting Elements Analysis of 22 Water-Deficit Responsive Nuclear Proteins Encoding Genes

We analyzed the main *cis*-acting elements in the 1,500 bp promoter region of 22 significantly up-regulated nuclear proteins encoding genes that showed a significant up-regulation in response to water deficit. In total, we identified 25 major *cis*-acting elements among these genes, which could be divided into five categories: metabolism related elements, environmental stress-related elements, hormones response elements, promoter related elements, and development related elements (Supplementary Table 6). Among them, *cis*-acting elements related to environmental stress and hormones response were particularly abundant. The environmental stress-related elements included LTR, antioxidant response element (ARE), MYC, MYB, WUN-motif, GC-motif, TC-rich repeats, and MBS. MYB in response to drought regulation and MYC in response to early drought were the most abundant. ARE and MBS (MYB binding site involved in drought induction) occurred frequently in some DAPs encoding genes such as PHD finger protein, TCP20 and E3 ubiquitin-protein ligase. The widely distributed *cis*-acting elements related to hormone response mainly included ABRE involved in ABA response and TGACG-motif involved in MeJA response. Additionally, some *cis*-acting elements related to transcription such as TATA-box and TGACG-box were also abundant.

### Transcriptional Expression Profiling by RNA-seq

**FIGURE 4:**
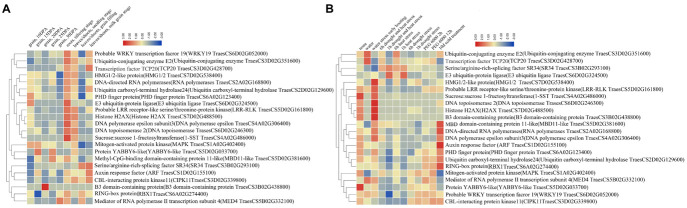
The relative expression profiles of the 38 up-regulated nuclear differentially accumulated proteins (DAPs) encoding genes. **(A)** In different development period of wheat different organs. **(B)** Different abiotic stresses. Red means high expression level, green means low expression level.

Using RNA-seq, we analyzed the transcriptional expression profiling of the 22 water-deficit responsive nuclear proteins encoding genes in different organs and growth periods as well as in response to various abiotic stresses. The publicly available RNA-seq database of bread wheat (var. Chinese Spring) allowed us to collect the relative expression levels of these genes. All the nuclear protein Uniprot ID was converted to wheat genetic ID by integrating it into GRAMENE, and then we submitted the gene ID to the bread wheat Chinese Spring public RNA-seq database to obtain the relative expression levels of 22 nuclear proteins encoding genes (Supplementary Table 7) and constructed the heat maps (Figure 4).

The expression profiling in different organs (root, leaf/shoot, and grain) showed that most DAPs encoding genes had a high expression level in root and leaf/shoot, and some genes had organ or growth stage expression preference (Figure 4A). In particular, the PHD finger protein and auxin response factor (ARF) encoding genes expressed in all organs. The DNA polymerases epsilon and sucrose 1-fructosyltransferase (1-SST) encoding genes showed an expression preference in roots, and five nuclear proteins encoding genes presented an expression preference in leaves/shoots, including *serine/arginine-rich-splicing factor SR34 isoform X3* (*SR34*), *ARF*, *CBL-interacting protein kinase 11* (*CIPK11*), *RING-box protein* (*RBX1*), and *mediator of RNA polymerase II transcription subunit 4* (*MED4*). In the grain, the PHD finger protein, E3 ubiquitin-protein ligase, and MAPK encoding genes showed a relatively high expression level at 10 or 15 DPA. In addition, some genes had relatively high expression in the root at the tilling stage, such as *E3 ubiquitin-protein ligase*, *DNA polymerases epsilon*, and *1-SST*. Others showed a relatively high expression level in the leaves/shoots at the milk grain stage, such as *TCP20*, *ARF*, and *CIPK11*.

Under abiotic stresses (water, drought, heat, and cold), most genes displayed distinct expression patterns (Figure 4B). The expression of ubiquitin-conjugating enzyme E2, TCP20, HMG1/2-like protein (HMG1/2), LRR-RLK, DNA topoisomerase, histone H2AX and B3 domain-containing protein encoding genes were up-regulated under water stress, while the expression of ARF and ubiquitin carboxy-terminal hydrolase encoding genes were up-regulated under cold treatment. In addition, some stress-responsive proteins encoding genes showed significant upregulation under multiple abiotic stresses, including SR34 and E3 ubiquitin-protein ligase under heat and drought stresses, MED4 encoding gene under water and PEG6000 stresses, MBD11, RNA polymerases and DNA polymerases epsilon encoding genes under water and cold stresses.

### Dynamic Transcriptional Expression Analysis by Real-Time Quantitative Polymerase Chain Reaction

**FIGURE 5:**
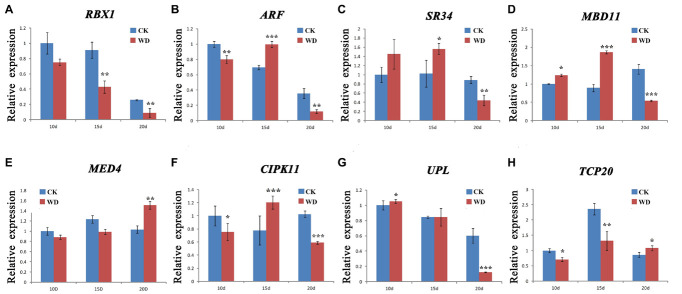
qRT-PCR analysis of eight representative differentially accumulated proteins (DAPs) encoding genes in nuclear-enriched fraction of Zhongmai 175 grain under water deficit. **(A)** RBX1, E3 ubiquitin-protein ligase RBX1; **(B)** ARF, Auxin response factor; **(C)** SR34, Serine/arginine-rich-splicing factor SR34 isoform X3; **(D)** MBD11, Methyl-CpG-binding domain-containing protein 11; **(E)** MED4, Mediator of RNA polymerase II transcription subunit 4; **(F)** CIPK11, CBL-interacting protein kinase 11; **(G)** UPL, putative E3 ubiquitin-protein ligase SINA-like 6; and **(H)** TCP20, transcription factor TCP20. Statistically significant differences were calculated based on an independent Student’s *t*-tests: *p < 0.05; **p < 0.01; and ***p < 0.001. CK, control; WD, water deficit.

We selected eight DAPs encoding genes closely related to wheat drought stress response to further detect their dynamic transcriptional expression patterns during grain development under water deficit by qRT-PCR. These DAPs encoding genes were significantly up-regulated or down-regulated under water-deficit conditions via preceding transcriptional analyses, including *RBX1*, *ARF*, *SR34*, *MBD11*, *MED4*, *CIPK11*, *putative E3 ubiquitin-protein ligase SINA-like 6* (*UPL*), and *TCP20*. Supplementary Table 8 lists the gene-specific primers used for qRT-PCR analysis and Figure 5 shows the results.

In general, eight nuclear DAPs encoding genes showed an up-down or down regulation expression during grain development under water-deficit treatment. Among them, five genes (*ARF*, *SR34*, *MBD11*, *MED4*, and *CIPK11*) were up-regulated at certain stages in response to water deficit while three (*RBX1*, *UPL*, and *TCP20*) were down-regulated. This was consistent with the results by RNA-seq (Figure 4). Compared to the protein levels, the transcriptional expression of four genes (*ARF, MBD11, SR34*, and *CIPK11*) showed a high consistency. However, the remaining four genes displayed a poor consistency between their transcription and translation expression as the previous report (Deng et al., 2018).

## Discussion

As sessile organisms, higher plants often face various environmental challenges during their growth and development. In the long-term evolutionary process, higher plants have formed a series of defense systems to cope with variable extreme environments, including environmental stress responses and gene expression regulation (Boyer, 1982). Transcription factors play a vital role in regulating gene expression, and many are associated with various abiotic stress responses (Zhu et al., 2015). In this study, we identified some transcription factor proteins with significant expression changes in response to water deficit, including TCP, NAC, and WRKY transcription factors (Supplementary Table 3).

T protein are plant-specific transcription factors characterized by the TCP domain, a motif encompassing a non-canonical basic-helix-loop-helix structure (Mar and Pilar, 2010). TCPs not only play an important role in controlling plant architecture (Aguilar-Martínez et al., 2007) and pollen development (Takeda et al., 2006), but they are also involved in responding to multiple abiotic stresses (Almeida et al., 2017; Liu et al., 2020). The expression level of *PeTCP10* in moso bamboo (*Phyllostachys edulis*) was induced by drought and ABA, and its overexpression significantly enhanced the tolerance of Arabidopsis and rice to drought stress (Liu et al., 2020). In Rice, *PCF2* was demonstrated to associate with salinity tolerance by directly targeting and positively regulating NHX1 that encodes a tonoplast-localized K^+^-Na^+^/H^+^ antiporter protein (Almeida et al., 2017). In addition, TCP21 and PCF6 were reported as targets of microRNA319 and positively regulate cold-stress tolerance (Wang et al., 2014). In the current study, we found that transcription factor TCP20 was significantly up-regulated in wheat developing grains under water-deficit conditions (Supplementary Table 3) and the peak expression level of the *TCP20* gene occurred at the grain milk stage (Figure 4A), indicating that TCP20 could play important roles in water-deficit response during wheat grain development.

The NAC proteins are one of the largest transcription factor family only discovered in plants (Mao et al., 2012). They play important roles in responding to various environmental stresses (Zhu et al., 2015). In Arabidopsis, the ectopic expression of *ANAC055*, *ANAC019*, or *ANAC072/RD26* was associated with enhanced tolerance to both salt and drought stresses (Tran et al., 2004). As reported, *ANAC096* functions as a positive regulator of drought stress response and cooperates with the ABF2 and ABF4 to activate the transcription of ABA-inducible genes *RD29A* in response to dehydration and osmotic stresses (Xu et al., 2015). In rice, drought and salt tolerance significantly increase according to overexpressing of *OsNAC10*, *OsNAC063*, *OsNAC5*, or *SANC1/OsNAC9* (Jeong et al., 2010, 2013; Fang et al., 2015; Shen et al., 2017). *SNAC3* confer heat and drought tolerance by modulating ROS homeostasis through enhancing ROS scavenging genes’ expression, including *R45* (RbohF, LOC_Os08g35210) and *R54* (Prx IIE2, LOC_Os02g09940; Fang et al., 2015). In *Pyrus betulifolia*, *beNAC1* improved stress tolerance by interacting with PbeDREB1 and PbeDREB2A to enhance the mRNA levels of some stress-associated genes (Jin et al., 2017). To date, an increasing number of NAC transcription factors in wheat were found to play crucial roles in a variety of abiotic stress responses, including *TaNAC2*, *TaNAC67*, *TaNAC29*, and *TaSNAC4* (Mao et al., 2012, 2014; Xu et al., 2015; Mei et al., 2021). In this study, quantitative grain nuclear proteome analysis identified three NAC domain-containing proteins, of which A0A3B6LX64 was significantly induced by water stress (Supplementary Table 3). This NAC domain-containing protein may be a new member of wheat NAC family involved in drought response.

WRKY transcription factor 19 (WRKY19, A0A3B6Q9R1) was also identified in the grain nuclear proteome (Supplementary Table 3). Plant WRKY transcription factors are downstream proteins of MAPK cascades, which are induced by ROS (H_2_O_2_) and in turn play an essential role in plant drought responses by activating transcription factors (Taj et al., 2011). In rice, *OsWRKY30* can be activated by MAPKs via phosphorylation, and its overexpression dramatically enhances drought tolerance (Shen et al., 2012). WRKY transcription factors are also key components in ABA signaling. *AtWRKY63* in Arabidopsis, activated by ABI5, activated downstream stress-inducible genes such as *RD29A* and *COR47* (Rushton et al., 2012). Our results showed that the protein accumulation level of WRKY19 (A0A3B6Q9R1) increased 2.244 times under water deficit (Supplementary Table 3) and the identified *cis*-acting elements in the promoter region of the *WRKY19* gene were predominantly associated with the stress- and hormone-responsive elements, including MYB, MYC, MBS, and ABRE and TGACG-motif (Supplementary Table 6). Moreover, the accumulation level of MAPK protein (A0A3B5Y6A9) was also significantly up-regulated under water-deficit condition (Supplementary Table 3). We speculated that *WRKY19* could be activated by MAPK protein, and then participated in water-deficit response via controlling downstream stress-related genes during wheat developing grains.

Most abiotic stresses can induce the changes of Ca^2+^ concentration, which are generally accepted as a secondary messenger to transduce the cellular responses to extracellular stimuli (Hofer and Brown, 2003; Kolukisaoglu et al., 2004). In plants, many Ca^2+^-sensing protein kinases are involved in the stress responses, including calcium-dependent protein kinases (Chandran et al., 2006) and suc non-fermentation-related kinases (SnRKs; Nozawa et al., 2001). CBL protein-interacting protein kinases (CIPKs) are members of the SnRK3 subfamily. In maize, overexpression of *ZmCIPK21* significantly enhances plant salt tolerance by decreasing accumulation of Na^+^ and allowing retention of relative high K^+^ (Chen et al., 2014). In rice, overexpressing *OsCIPK03, OsCIPK12*, and *OsCIPK15* showed a higher tolerance to cold, drought and salt stress, respectively. Meanwhile, researchers have found a higher content of proline and soluble sugar in *OsCIPK03*- and *OsCIPK12*-overexpressing transgenic plants (Xiang et al., 2007). Recent study has found that *TaCIPK27* dramatically enhanced drought tolerance of Arabidopsis by regulating ABA-related genes’ expression such as *ABI1*, *RD29B* and drought-related genes such as *DREB1B* and *DREB2A* (Wang et al., 2018). In this study, the abundance of CIPK11 (A0A3B6H0R7) protein was increased by 2.015 times and its transcription level was also significantly up-regulated at 15 DPA under water deficit (Figure 5). These results indicate that CIPK11 may contribute to water-deficit response by regulating the expression of downstream stress-related genes during wheat grain development.

Histone acetylation is a dynamic process that affects chromatin structure and regulates gene expression. This process is regulated by two types of enzymes: histone acetyltransferases and histone deacetylases (HDAs). In plants, HDACs could be classified into four different subfamilies: reduced potassium dependency protein 3 (RPD3), histone deacetylase 1 protein (HDA1), HD2-like proteins (HD2) and the silent information regulator protein 2 (SIR2; Lusser et al., 1997; Ruijter et al., 2003). Previous studies reported that Arabidopsis HD2C, a HD2-type HDA, was involved in the ABA and salt-stress response by interacting with HDA6 (RPD3-type HDA) and modulating the expression of stress-responsive genes such as *ABI1*, *ABI2*, and *AtERF4* (Luo et al., 2012). The transgenic Arabidopsis with *BdHD1* overexpression displayed a hypersensitive phenotype to ABA and exhibited better survival under drought conditions (Song et al., 2019). These results indicated that HDAs were closely associated with response to abiotic stress. Recently, lysine acetylproteome profiling under wheat water deficit found that many acetylated proteins are involved in grain development and starch biosynthesis (Zhu et al., 2018). We also found that the histone deacetylase complex subunit SAP18 (A0A3B6PGM4) was up-regulated under water deficit (Supplementary Table 3), which may positively regulate drought responses by activating the expression of stress-responsive genes via regulating histone acetylation levels during grain development.

The ubiquitin-proteasome system is mechanistically conserved in eukaryotes consisting of an intricate collection of enzymes and enzyme complexes that conjugate ubiquitin to target proteins and facilitate the degradation of ubiquitinated proteins (Vierstra, 2009). Target proteins are ubiquitinated via an ATP-dependent reaction cascade that generally involves the sequential action of E1 ubiquitin-activating enzyme, E2 ubiquitin-conjugating enzyme, and E3 ubiquitin ligase. In plants, both the E2 ubiquitin-conjugating enzyme and the E3 ubiquitin ligase are pivotal in responding to drought stress (Lyzenga and Stone, 2012; Shu and Yang, 2017). In Arabidopsis, overexpression of E2 ubiquitin-conjugating enzyme, soybean *GmUBC2*, peanut *AhUBC2* and mung bean *VrUBC1* significantly enhanced the drought tolerance through modulating abiotic stress-responsive gene expression (Zhou et al., 2010; Wan et al., 2011; Chung et al., 2013). In addition, SDIR1 (SALT- AND DROUGHT-INDUCED RING FINGER 1), a RING type E3 ligase, serves as a positive regulator of ABA signaling by promoting the transcription of *ABI3* and *ABI5* genes and the overexpression of *SDIR1* consistently enhanced plant drought tolerance (Zhang et al., 2007). Furthermore, another RING type E3 ligase AIRP1 (ABA-insensitive RING protein 1), also functions as a positive regulator in the ABA dependent drought responses, and *AIRP1*-overexpresing transgenic plants showed a drought tolerant phenotype while the *atairp1* mutant was highly susceptible to water-deficit condition (Ryu et al., 2010). In this study, we found that ubiquitin-conjugating enzyme E2 (A0A3B6PIG4) and E3 ubiquitin-protein ligase RBX1 (W5GNW2) were, respectively, increased by 2.011 and 2.459 times, indicating that the process of ubiquitination may play important roles in wheat grain responding to drought stress during development.

**FIGURE 6:**
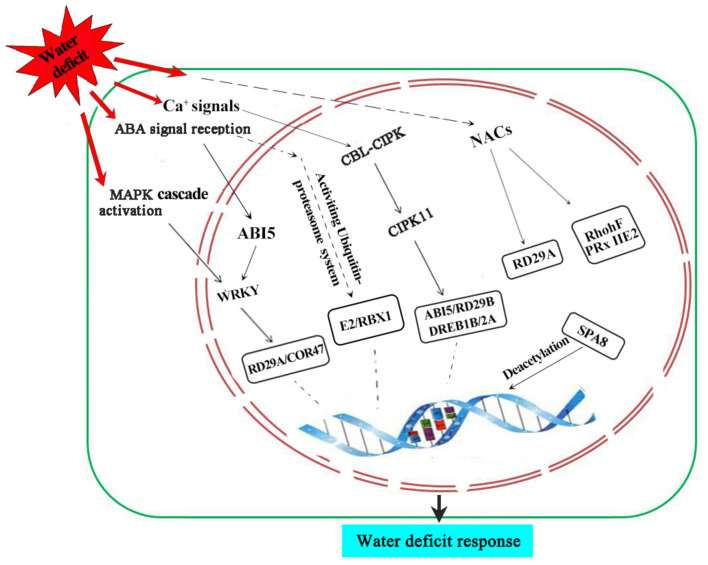
A putative synergistic responsive network of the wheat grain proteome to water-deficit stress. SnRK2, suc non-fermentation-related kinases; CIPK11, CBL protein-interacting protein kinase 11.

Based on the results of this study and previous reports, we propose a putative pathway of wheat grain nuclear subproteome to respond to water deficit (Figure 6). Under water deficit, ABA and Ca^2+^ signal induces the expression of transcription factors such as *TCP20*, *WRKY*, *NAC*, and *ARF*, thereby activating the expression of stress-related genes to cope with water-deficit stress. A MAPK cascade signaling system and CBL-CIPK signaling pathway of ABA-dependent endow plants with resistance to water deficits. In addition, some proteins involved in the process of chromatin remodeling and ubiquitin-proteasome system were also activated to adapt or resist water deficit during wheat grain development.

## Conclusion

Label-free quantitative proteomic analysis identified 398 water-deficit responsive DAPs in the cell nucleus subproteome of wheat developing grains. Among them, 146 up-regulated DAPs were mainly involved in stress response and oxidation-reduction process while 252 down-regulated DAPs mainly participated in translation, the cellular amino metabolic process, and the oxidation-reduction process. In particular, the promoter region of 22 up-regulated DAPs encoding genes under water deficit contained abundant *cis*-acting elements related to environmental stresses and hormones response such as ABRE, ARE, MYB, and MYC. We detected the transcriptional expression profiling of these DAPs encoding genes in different organs, developmental stages and abiotic stresses by using RNA-seq and qRT-PCR. We proposed a putative metabolic pathway of wheat grain nuclear subproteome in response to water deficit, thereby providing new evidence from a subcellular proteome level for further understanding the molecular mechanisms of plant drought stress response.

## Data Availability Statement

The datasets presented in this study can be found in online repositories. The names of the repository/repositories and accession number(s) can be found below: http://www.proteomexchange.org/, PXD021315.

## Author Contributions

TL, DZ, and ZH performed most of the experiments, data analysis, and wrote the manuscript. JZ formed part of the experiments and data collection. MZ and YY designed and supervised the experiments. All authors contributed to the article and approved the submitted version.

## Conflict of Interest

The authors declare that the research was conducted in the absence of any commercial or financial relationships that could be construed as a potential conflict of interest.

## Publisher’s Note

All claims expressed in this article are solely those of the authors and do not necessarily represent those of their affiliated organizations, or those of the publisher, the editors and the reviewers. Any product that may be evaluated in this article, or claim that may be made by its manufacturer, is not guaranteed or endorsed by the publisher.

## Footnotes

1
http://www.genscript.com/wolf-psort.html

2
http://cello.life.nctu.edu.tw/

3
http://www.csbio.sjtu.edu.cn/bioinf/plant-multi/

4
https://www.uniprot.org/

5
http://bioinformatics.psb.ugent.be/webtools/plantcare/html/

6
http://ensembl.gramene.org/

7
http://www.wheat-expression.com/

8
www.bioinformatics.nl/cgi-bin/primer3plus/primer3plus.cgi

## Supplementary Material

Supplementary Figure 1 Morphological observation of the nucleus extracted from wheat developing grains by using Hoechst staining.

Supplementary Table 1 The list of peptides identified from extracted nucleus of wheat developing grains under water deficit.

Supplementary Table 2 The list of unique proteins identified from extracted nucleus of wheat developing grains.

Supplementary Table 3 Subcellular localization analysis of differentially accumulated proteins (DAPs) identified from extracted nucleus of wheat developing grains under water deficit.

Supplementary Table 4 List of primers used for gene cloning and vector constructions.

Supplementary Table 5 GO annotation of nucleus localized differentially accumulated proteins (DAPs) identified from extracted nucleus of wheat developing grains under water deficit.

Supplementary Table 6 The *cis*-acting elements in the 1,500 bp upstream promoter regions of 22 significantly up-regulated nuclear proteins encoding genes under water deficit.

Supplementary Table 7 Transcription expression profile of 22 water deficit responsive nuclear proteins encoding genes in different tissues and developing stages and abiotic stresses based on RNA-seq data.

Supplementary Table 8 Sequence of specific primers for qRT-PCR analysis.
