# Supplementary figures and images for: Label-Free Quantitative Proteome Analysis Reveals the Underlying Mechanisms of Grain Nuclear Proteins Involved in Wheat Water-Deficit Response
Source: Front Plant Sci. 2021 Oct 25;12:748487. doi: 10.3389/fpls.2021.748487 (PMC8572964; doi:10.3389/fpls.2021.748487)

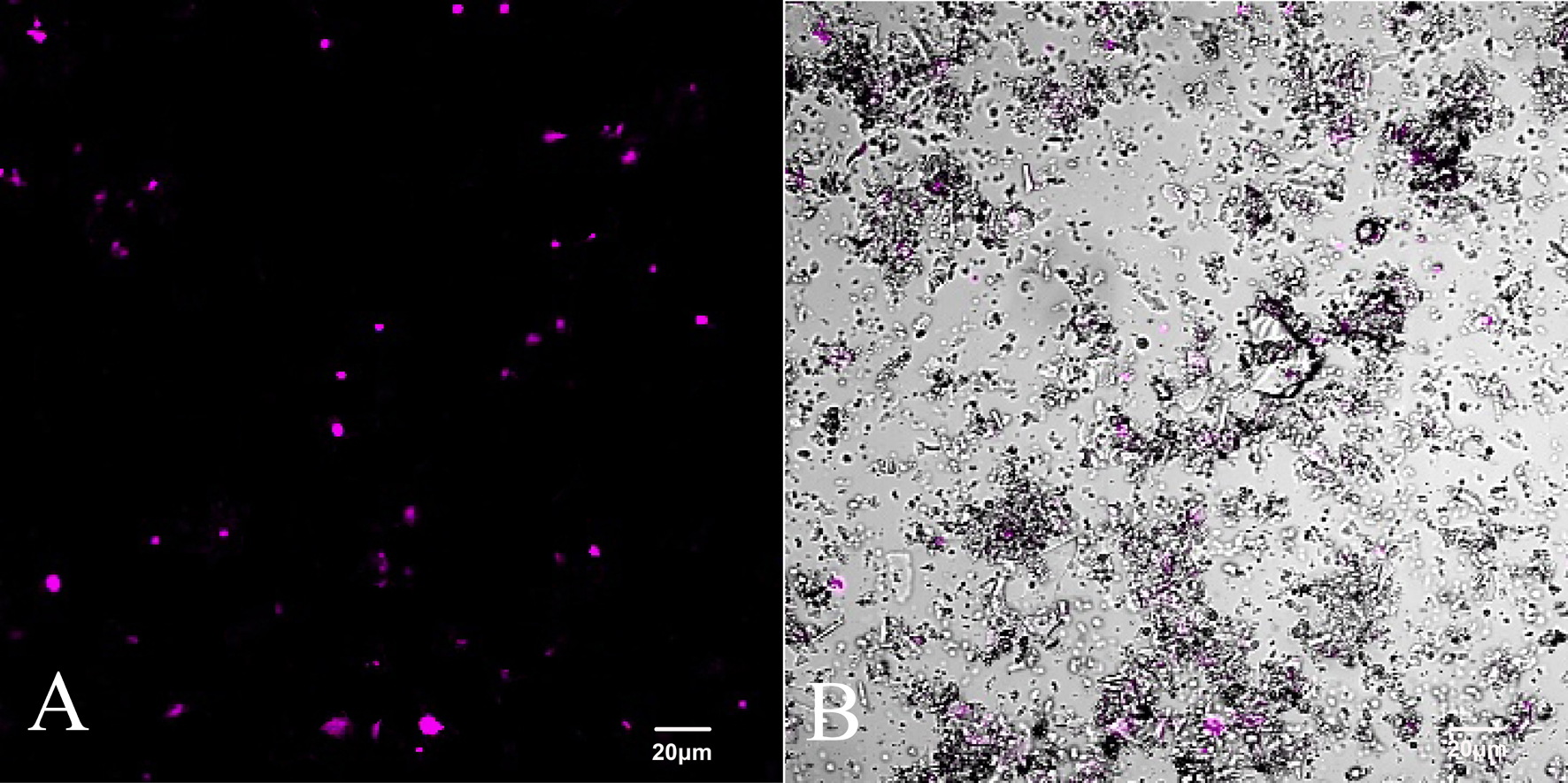

Supplement: Supplementary Figure 1 — Morphological observation of the nucleus extracted from wheat developing grains by using Hoechst staining. [file Image_1.JPEG]
